# Hybridization on DNA‐Coated Ultrasmall Gold Nanoparticles (2 nm)

**DOI:** 10.1002/chem.202502421

**Published:** 2025-10-09

**Authors:** Jonas Sager, Kateryna Loza, Oleg Prymak, Marc Heggen, Alexander Huber, Jens Voskuhl, Cristiano L. P. Oliveira, Matthias Epple

**Affiliations:** ^1^ Inorganic Chemistry and Center for Nanointegration Duisburg‐Essen (CENIDE) University of Duisburg‐Essen 45141 Essen Germany; ^2^ Ernst Ruska Centre for Microscopy and Spectroscopy with Electrons Forschungszentrum Jülich 52428 Jülich Germany; ^3^ Organic Chemistry and Center for Nanointegration Duisburg‐Essen (CENIDE) University of Duisburg‐Essen 45141 Essen Germany; ^4^ Institute of Physics University of São Paulo São Paulo 05508‐090 Brazil

**Keywords:** DNA, fluorescence, hybridization, nanoparticles, oligonucleotides

## Abstract

Ultrasmall gold nanoparticles were functionalized with covalently attached DNA strands of 20 or 30 nucleotides. This was achieved via click chemistry with alkyne‐terminated DNA and azide‐terminated gold nanoparticles. The particles were characterized by high‐resolution transmission electron microscopy, UV‐Vis spectroscopy, fluorescence spectroscopy, and small‐angle X‐ray scattering. The DNA strands were fluorescently labelled with either FAM or Cy3, permitting their detection and quantification on the nanoparticle surface. Complementary DNA strands were attached to the nanoparticles via hybridization. The connection of gold nanoparticles by complementary DNA strands was also demonstrated. In‐situ fluorescence spectroscopy confirmed the hybridization at ambient temperature and the melting of the DNA strands at elevated temperature. The hybridization was confirmed by fluorescence spectroscopy with the FRET effect. This opens broad possibilities for the noncovalent functionalization of ultrasmall nanoparticles.

## Graphical abstract

DNA‐conjugated ultrasmall gold nanoparticles (2 nm) hybridize with complementary DNA strands to form dimers. Covalent attachment via click chemistry preserves the DNA functionality, creating building blocks for supramolecular chemistry and controlled nanoparticle assembly.

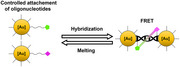

## 1 Introduction

The selective and stable hybridization of complementary DNA strands is one of the most frequently applied examples of noncovalent interactions that are used for self‐organization. It has been widely used to arrange and connect molecules that carry complementary DNA strands. For nanoparticle self‐assembly, this has been extensively elaborated by Mirkin et al. to prepare organized assemblies of nanoparticles arranged by complementary DNA strands.^[^ ^1^, ^2^ ^]^ Upon heating, the DNA strands can disconnect, a process that is usually denoted as melting. This allows a thermal switching to cleave the bonds between prearranged molecules or particles. Another noteworthy example of hybridized DNA strands is DNA origami where structures of remarkable complexity have been designed and realized.^[^ ^3^, ^4^ ^]^ Small‐angle X‐ray scattering has been successfully applied to supramolecular DNA structures ^[^ ^5^, ^6^, ^7^, ^8^ ^]^ including DNA origami.^[^ ^9^, ^10^, ^11^ ^]^

The covalent functionalization of ultrasmall nanoparticles with DNA has not yet been reported, although there are some reports on nucleic‐acid functionalized larger nanoparticles.^[^ ^12^, ^13^, ^14^ ^]^ Ultrasmall nanoparticles, unlike the larger plasmonic nanoparticles, do not quench fluorescence^[^ ^15^ ^]^ and can also be analysed by NMR spectroscopy in dispersion.^[^ ^16^, ^17^, ^18^ ^]^ Their small diameter of 1–3 nm places them at the border between atom‐sharp metalloid clusters and metallic nanoparticles.^[^ ^19^, ^20^ ^]^ Typically, they consist of several hundred metal atoms. Their surface is protected by suitable capping ligands, usually attached by the strong sulphur‐gold bond via thiol groups on the ligands.^[^ ^21^ ^]^ Due to the high surface curvature, a 2 nm particle can carry one hundred or more ligands, depending on the spatial requirements of the ligand.^[^ ^19^ ^]^

We have reported earlier how glutathione‐coated ultrasmall nanoparticles can be converted into azide‐coated nanoparticles with an azide transfer reagent that converts the amine groups of glutathione into azide groups.^[^ ^22^ ^]^ This opens the pathway for copper‐catalysed azide‐alkyne cycloaddition (CuAAC), making it possible to attach all kinds of alkyne‐carrying molecules to the nanoparticle surface. This reaction avoids a degradation of the ligands during the comparatively harsh reaction conditions during the synthesis in the presence of the strongly oxidizing metal cations (Au^3+^) and the strongly reducing NaBH_4_. The ligands are firmly attached by the strong covalent bond. We have attached various dyes, receptor molecules, and also nucleic acids like siRNA to the surface of ultrasmall nanoparticles via click chemistry.^[^ ^23^, ^24^, ^25^, ^26^, ^27^, ^28^ ^]^

Here we report how ultrasmall nanoparticles were surface‐functionalized by different DNA strands. The DNA strands carried dyes at one end (FAM or Cy3), thereby rendering the nanoparticles fluorescent. The quenching by the metal was assessed by measuring the quantum efficiency. The successful hybridization with a complementary dye‐labelled DNA strand was shown and quantified by UV‐Vis spectroscopy. Finally, the transfer of the electron excitation from one dye to another via Förster Resonance Energy Transfer (FRET) confirmed the proximity and hybridization of complementary DNA strands.

Finally, we demonstrate how nanoparticles can be connected via complementary DNA strands.

## 2 Materials and Methods

### 2.1 Reagents

Hydrochloric acid (HCl, 37%), nitric acid (HNO_3_, 67%), and sodium hydroxide (NaOH, 1 M) were obtained from Bernd Kraft (Duisburg, Germany). Sodium borohydride (NaBH_4_, 99%), 10 kDa and 30 kDa spin filters were obtained from Merck (Darmstadt, Germany). Glutathione (GSH, 98%), potassium carbonate (K_2_CO_3_, 99%), copper sulphate (CuSO_4_, 98%), tris(3‐hydroxypropyl‐triazolylmethyl) amine (THPTA, 96%) and sodium ascorbate (99%) were obtained from Fisher Scientific (Geel, Belgium). Aminoguanidine hydrogen carbonate (98%) was obtained from Alfa Aesar (Kandel, Germany). Sucrose was obtained from VWR Chemicals (Langenfeld, Germany). Cy3‐alkyne (95%) was obtained from Lumiprobe (Hannover, Germany). Dodecane and the PVC nanoparticle calibration dispersion (Lot#149, 1.385 g L^−1^ were obtained from CPS Instruments Inc. (Oosterhout, The Netherlands). The oligonucleotides were obtained from Integrated DNA Technologies (IDT, Leuven, Belgium). All oligonucleotides were modified at the 5′ end with an octyne group, 55OctdU: 5‐OctadiynyldU, which provided the alkyne functionality for CuAAC click chemistry, followed by the spacer group iSpC3: Int C3 spacer phosphoramidite. The opposite 3′ end was labelled with a fluorophore (Cy3 or FAM) for detection by UV/Vis spectroscopy and fluorescence spectroscopy. The labels were 36‐FAM (fluorescein) and 3Cy3Sp (Cy3). The structures of these modifications according the Integrated DNA Technologies are given in the Supporting Information (Figure S6).

Ultrapure water (Purelab Ultra instrument, 18.2 MΩ, ELGA) was used in all cases. All glassware was cleaned by boiling once with concentrated nitric acid, followed by rinsing with water.

### 2.2 Electron Microscopy

High‐resolution transmission electron microscopy (HRTEM) was performed on nanoparticles deposited onto ultrathin amorphous carbon films supported on copper mesh grids with a Cs‐corrected FEI transmission electron microscope (Thermo Fisher Scientific), operated at 300 kV.^[^ ^29^ ^]^

### 2.3 Small‐angle X‐ray Scattering (SAXS)

SAXS data were performed on a laboratory‐based instrument Xenocs‐Xeuss 2.0 at the EMUSAXS centre at the Institute of Physics, University of São Paulo. This machine is equipped with a microfocus source Genix3D (Cu Kα; *λ* = 1.5418 Å), Fox3D focusing mirrors, and two sets of scatterless slits. The 2D scattering images were collected on a Dectris‐Pilatus 300k detector. The azimuthal integrations were performed with the program package Fit2D.^[^ ^30^ ^]^ As a result, 1D curves of the scattering intensity as a function of the modulus of the reciprocal space scattering vector *q* were obtained. *q* is defined as q=4πsin(θ)/λ, where 2θ is the scattering angle. The liquid samples were placed on homemade sample holders composed of borosilicate glass capillaries (1.5 mm in diameter) glued on stainless steel cases and closed with rubber caps. Therefore, the measurements could be performed in vacuum. Since these sample holders could be washed and rinsed, the samples and the corresponding blanks were measured under exactly the same conditions, allowing a better data treatment.^[^ ^31^ ^]^ Data treatment and uncertainties estimations were performed with the program package SuperSAXS.^[^ ^32^ ^]^ The sample to detector distance was 580 mm, giving a useful range of 0.012 < *q* < 0.7 Å^−1^. Plain water was used as blank for the measurements as well as for normalization to an absolute scale.

The data analysis of the obtained scattering intensities was performed by several model approaches. For the monodisperse systems the aggregated polydisperse hard spheres model (APS) described in previous works was used.^[^ ^6^ ^]^ In this model, a polydisperse system of spheres with average radius *R* and polydispersity *σ*, given by a form factor *P*(*q*)_sph_ is assumed. The spheres can form large aggregates with overall radius of gyration *RG*_agg_, which is included in the structure factor *S*(*q*)_agg_. Also, since the spheres are hard, interaction effects that are represented by a hard spheres structure factor *S*(*q*)_HS_ can be computed. The final theoretical scattering intensity is given by eq. (1)

(1) $$I\;\left(q\right)=S_{C}\;\;S{\left(q\right)}_{agg}\;S{\left(q\right)}_{HS}\;P{\left(q\right)}_{sph}+back$$

where *S_C_* is an overall scale factor and *back* a constant background. All mathematical details can be found in previous articles.^[^ ^33^ ^]^

### 2.4 Differential Centrifugal Sedimentation (DCS)

Differential centrifugal sedimentation was performed with a DC24000 instrument (CPS Instruments). The disc was accelerated to a rotational speed of 24,000 rpm, followed by the injection of a sucrose gradient solution. The gradient was prepared by layering 24 wt% to 8 wt% sucrose solutions in water. To prevent evaporation, the gradient solution was overlaid with 0.5 mL dodecane. Calibration was performed with a dispersion of stabilized polyvinyl chloride (PVC) particles with a hydrodynamic diameter of 483 nm obtained from CPS.

### 2.5 UV‐Vis‐Spectroscopy

UV‐Vis spectra were recorded at room temperature with a Cary 300 spectrometer (Varian) from 200 to 800 nm. Measurements were performed in Suprasil quartz cuvettes with a sample volume of 600 µL and a gold concentration of 10 µg mL^−1^. The background signal of pure water was used for correction. The amounts of substance were 2.07 nmol for 20 nt and 1.45 nmol for 30 nt DNA strands, respectively.

### 2.6 Fluorescence Spectroscopy / Quantum Yield Measurements

Fluorescence spectroscopy was performed in a fluorescence cuvette (600 µL) with a Cary Eclipse spectrometer (Agilent Technologies). FAM was excited at 490 nm, and Cy3 was excited at 525 nm. For temperature‐dependent studies, a Cary Single Cell Peltier Accessory Setup (Agilent Technologies) was used. Absolute photoluminescence quantum yields were determined with an RF‐6000 spectrofluorometer from Shimadzu equipped with an ISR‐6000 integrating sphere. The amounts of substance were 2.07 nmol for 20 nt and 1.45 nmol for 30 nt DNA strands, respectively.

### 2.7 Atomic Absorption Spectroscopy (AAS)

To determine the gold concentration, 10 µL of a gold nanoparticle dispersion was dissolved in 190 µL *aqua regia* and subsequently diluted with water to a total volume of 5 mL. The analysis was performed with an M‐series spectrometer (ThermoElectron Corporation, USA) with a graphite furnace operated in accordance with DIN EN ISO/IEC 17 025:2005.

### 2.8 Synthesis of Azide‐terminated Gold Nanoparticles

Tetrachloridoauric acid was prepared by dissolution of elemental gold in boiling *aqua regia*. Glutathione‐functionalized nanoparticles (Au‐GSH) were synthesized as previously reported by a modified Brust‐Schiffrin synthesis via reduction of tetrachloridoauric acid with NaBH_4_ in the presence of glutathione.^[^ ^22^ ^]^ The particles were isolated and purified by spin filtration at pH 13 (adjusted with NaOH) with 10 kDa Amicon spin filters (4000 rpm, 2500 g, 25 min). Au‐GSH nanoparticles were transformed into azide‐terminated Au‐GSH‐N_3_ nanoparticles by conversion of amine groups from glutathione to azide by an azide transfer reagent as described earlier.^[^ ^22^ ^]^ The Au‐GSH‐N_3_ nanoparticles were isolated and purified by spin filtration as with Au‐GSH nanoparticles. Each gold nanoparticle carried about 125 GSH molecules before azidation and about 118 azide groups after azidation.^[^ ^22^ ^]^ A spherical 2 nm gold nanoparticle contains approximately 250 gold atoms,^[^ ^34^ ^]^ resulting in a stoichiometry of about Au_250_GSH_125_. The molecular mass of the Au‐GSH‐NP nanoparticles was therefore estimated as the sum of 49,250 g mol^−1^ Au and 38,288 g mol^−1^ GSH, giving a molecular weight of about 88.5 kDa. Accordingly, the gold content in each Au‐GSH nanoparticle was approximately 56 wt%.

### 2.9 Conjugation of DNA to the Surface of Nanoparticles

Azide‐terminated DNA oligonucleotides were attached to Au‐GSH‐N_3_ nanoparticles by copper‐catalysed azide‐alkyne cycloaddition (CuAAC), following earlier protocols where azide‐terminated siRNA was conjugated to Au‐GSH‐N_3_ nanoparticles.^[^ ^28^ ^]^

An aqueous solution containing Cu^2+^, tris((1‐hydroxypropyl‐1H‐1,2,3‐triazol‐4‐yl)methyl)amine (THPTA), and aminoguanidine bicarbonate was prepared by dissolving CuSO_4_ (10 µmol, 1.6 mg), THPTA (500 µmol, 217 mg), and aminoguanidine bicarbonate (10 µmol, 1.4 mg) in 10 mL water.

Au‐OligoFAM‐20nt: Au‐GSH‐N_3_ nanoparticles (10.8 nmol Au‐GSH‐N_3_, 500 µg Au) were dispersed in 4 mL water. OligoFAM‐20 nt (16.6 nmol, 120 µg), dissolved in 200 µL water, was added. Then, 95 µL of the Cu^2+^ solution described above were added. To initiate the click reaction, sodium ascorbate (250 µmol, 50 µg in 75 µL water) was added. After 72 h stirring at ambient temperature, the reaction mixture was diluted with 20 mL water and washed four times by spin filtration (30 kDa Amicon spin filter) at 4000 rpm (2500 g) for 15 min with water. In preliminary experiments we confirmed that unbound DNA (20 nt and 30 nt) passes through the spin filter under these conditions, i.e., it is separated from the nanoparticles that carry DNA. This also permitted to assess the efficiency of DNA clicking via UV spectroscopy which was usually very high, i.e., above 50% with respect to DNA.

Au‐OligoCy3‐20nt: Au‐GSH‐N_3_ nanoparticles (10.8 nmol Au‐GSH‐N_3_, 500 µg Au) were dispersed in 4 mL water. OligoCy3‐20 nt (17 nmol, 123 µg), dissolved in 200 µL water, was added. Then, 95 µL of the Cu^2+^ solution described above were added. To initiate the click reaction, sodium ascorbate (250 µmol, 50 µg in 75 µL water) was added. After 72 h stirring, the particles were isolated as described above.

Au‐OligoFAM‐30nt: Au‐GSH‐N_3_ nanoparticles (8.64 nmol Au‐GSH‐N_3_, 400 µg Au) were dispersed in 4 mL water. OligoFAM‐30 nt (18.9 nmol, 195 µg), dissolved in 200 µL water, was added. Then, 76 µL of the Cu^2+^ solution described above were added. To initiate the click reaction, sodium ascorbate (200 µmol, 40 µg in 60 µL water) was added. After 72 h stirring, the particles were isolated as described above.

Au‐OligoCy3‐30nt: Au‐GSH‐N_3_ nanoparticles (13 nmol Au‐GSH‐N_3_, 600 µg Au) were dispersed in 4 mL water. OligoCy3‐30 nt (25.3 nmol, 175 µg), dissolved in 200 µL water, was added. Then, 116 µL of the Cu^2+^ solution described above were added. To initiate the click reaction, sodium ascorbate (300 µmol, 60 µg in 90 µL water) was added. After 72 h stirring, the particles were isolated as described above.

Au‐OligoCy3‐30ntrev: Au‐GSH‐N_3_ nanoparticles (13 nmol Au‐GSH‐N_3_, 600 µg Au) were dispersed in 4 mL water. OligoCy3‐30ntrev (16.5 nmol, 171 µg), dissolved in 200 µL water, was added. Then, 116 µL of the Cu^2+^ solution described above were added. To initiate the click reaction, sodium ascorbate (300 µmol, 60 µg in 90 µL water) was added. After 72 h stirring, the particles were isolated as described above.

### 2.10 Annealing and Hybridization

The melting temperature (*T*_m_) of the oligonucleotides was 51.0 °C of 20 nt oligonucleotides and 62.8 °C for 30 nt oligonucleotides according to the manufacturer. In all cases, 2∙15 µg of each oligonucleotide were used. This corresponds to 2.07 nmol for 20 nt and 1.45 nmol for 30 nt DNA strands. Two complementary DNA strands (alone or attached to gold nanoparticles) were dissolved in 3 mL water with a pH of 8.5 that was adjusted with 1 mM NaOH. The solution was homogenized by vortex mixing and then heated to *T*_m_, maintained at this temperature for 10 min, and then freely cooled to room temperature. This cycle was repeated three times.

## 3 Results and Discussion

Ultrasmall gold nanoparticles were covalently functionalized with DNA strands. For this, glutathione‐coated ultrasmall gold nanoparticles were converted into azide‐terminated nanoparticles with an azide transfer reagent.^[^ ^22^ ^]^ Short (20 nucleotides) and long (30 nucleotides) DNA strands were attached by CuAAC click chemistry. The nucleotides were fluorescently labelled with either FAM or Cy3 on the 3′ end and with an octyne group for clicking at the 5′ end. All sequences, melting temperatures (*T*_m_), and molecular weights of the oligonucleotides are given in Table 1. DNA‐carrying ultrasmall gold nanoparticles can be hybridized with complementary DNA strands. The melting temperatures of 51.0 °C (20 nt) and 62.8 °C (30 nt) ensured a stable hybridization at ambient temperature but permitted to observe the melting in‐situ at elevated temperature by fluorescence spectroscopy. The dyes for labelling were chosen to permit an assessment of the proximity of conjugated DNA strands via the Foerster Resonance Energy Transfer (FRET) effect.^[^ ^35^ ^]^

**Table 1:** Properties of the oligonucleotides used in the conjugation studies (see experimental part and Figure S6 for the conjugated moieties for click chemistry and fluorescent labelling).

Oligonucleotide	Sequence	Melting temperature *T*_m_ / °C	Molecular weight *M* / g mol^−1^
OligoFAM‐20nt	5“‐/55OctdU//iSpC3/TA CGA GTT GAG AAT CCT GAA/36‐FAM/‐3”	51.0	7267
OligoCy3‐20nt	5“‐/55OctdU//iSpC3/TT CAG GAT TCT CAA CTC GTA/3Cy3sp/‐3”	51.0	7244
OligoFAM‐30nt	5“‐/55OctdU//iSpC3/TA CGA GTT GAG AAT CCT GAA TGC GCG TTC A /36‐FAM/‐3”	62.8	10348
OligoCy3‐30nt	5“‐/55OctdU//iSpC3/TG AAC GCG CAT TCA GGA TTC TCA ACT CGT A/3Cy3sp/‐3”	62.8	10343
OligoCy3‐30ntrev	5“‐/55OctdU//iSpC3/AT GCT CAA CTC TTA GGA CTT ACG CGC AAG T/3Cy3sp/‐3”	62.8	10343

**Figure 1:**
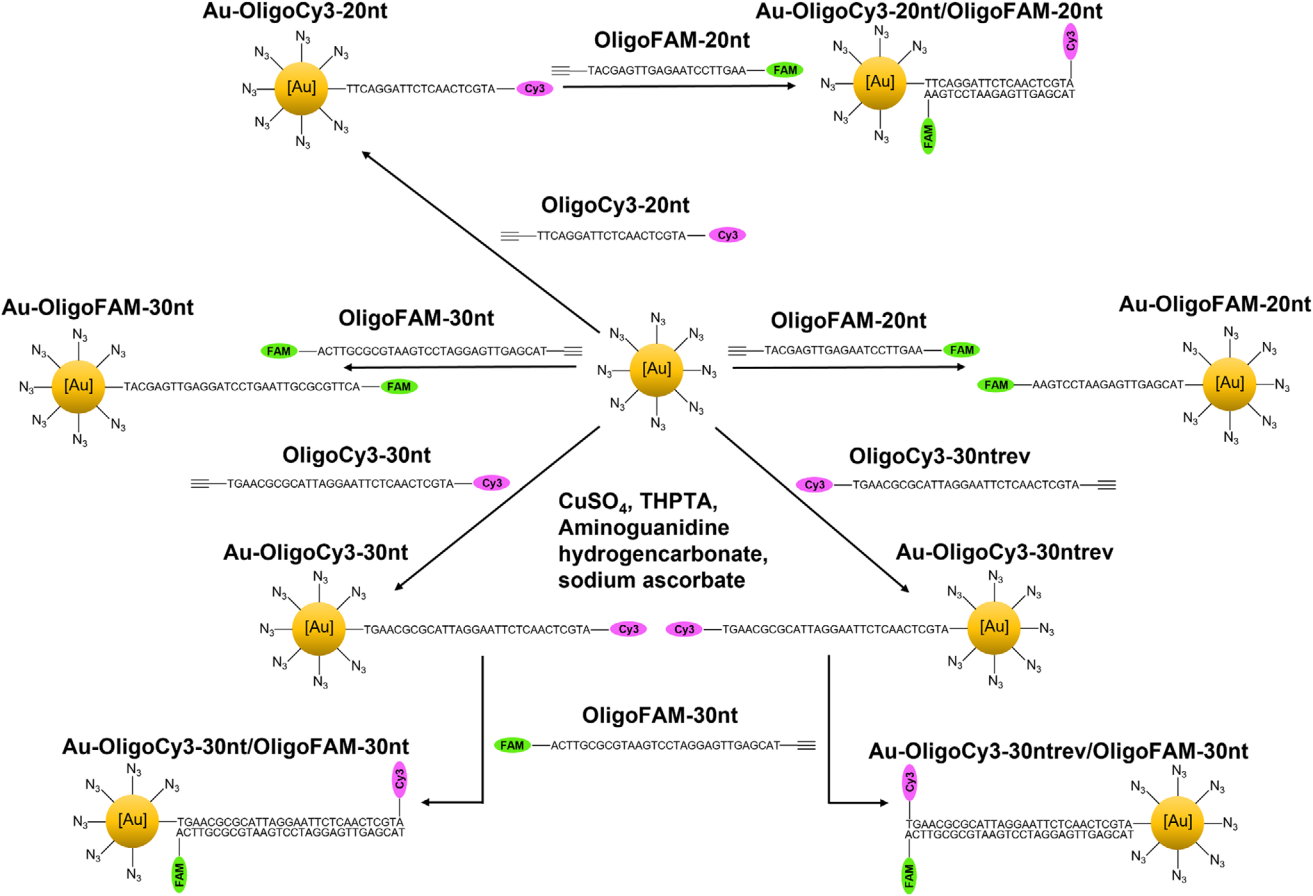
Schematic representation of the synthetic pathways and the covalent conjugation of DNA to nanoparticles and of the hybridization with complementary DNA strands. The DNA strands were fluorescently labelled with either FAM or Cy3 to permit their detection and quantification, before and after conjugation and hybridization.

Figure 1 gives an overview of all nanoparticles and all hybridized systems. Most DNA duplexes carried the dye at opposite ends, but for comparison, a reverse sequence was also used where the dyes were at the same end of the DNA duplex (denoted as “rev”).

**Figure 2:**
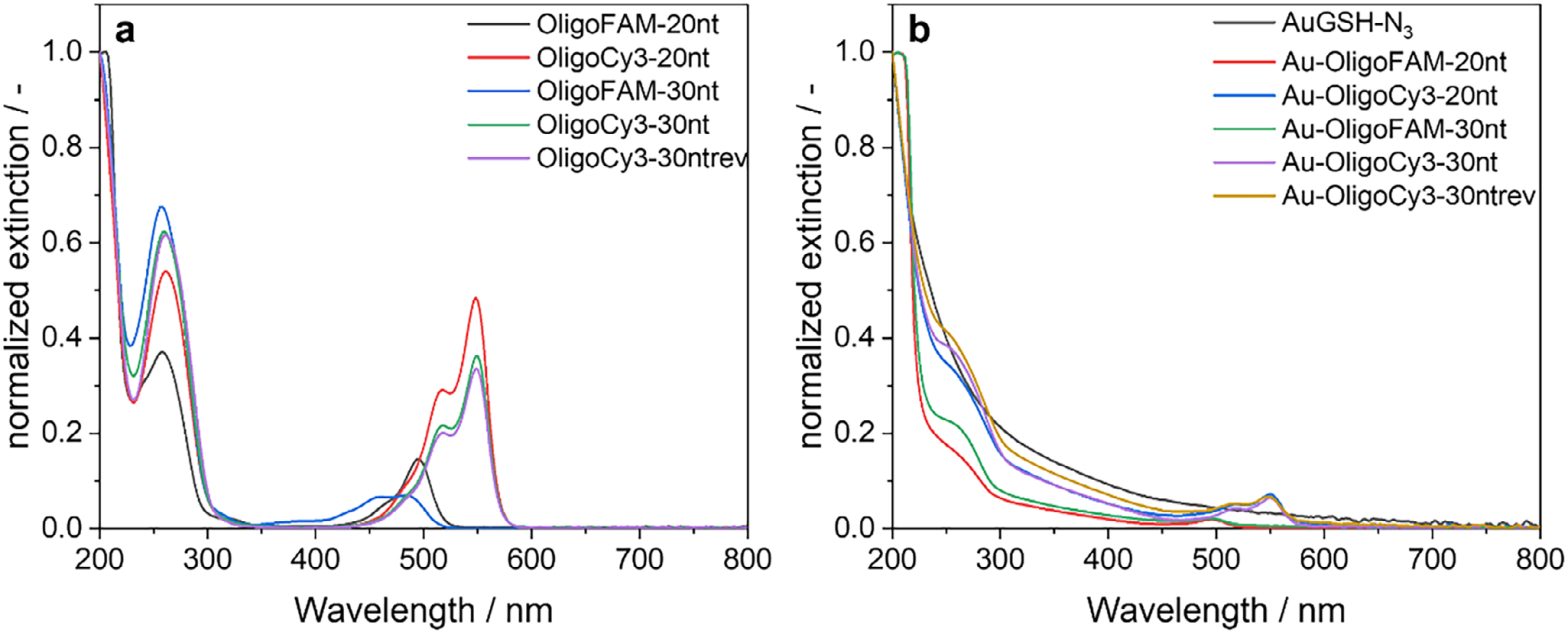
**a**) Normalized UV‐Vis spectra (25 °C in water) of the nonconjugated DNA strands OligoFAM‐20 nt (**black**), OligoCy3‐20 nt (**red**), OligoFAM‐30 nt (**blue**), OligoCy3‐30 nt (**green**), and OligoCy3‐30ntrev (**purple**). **b**) Normalized UV‐Vis spectra of the nanoparticles Au‐GSH‐N3 (**black**), Au‐OligoFAM‐20 nt **(red**), Au‐OligoC3‐20 nt (**blue**), Au‐OligoFAM‐30 nt (**green**), Au‐OligoCy3‐30 nt (**purple**), and Au‐OligoCy3‐30ntrev (**gold**). The absorption of FAM has a maximum at 495 nm, the absorption of Cy3 at 550 nm. All samples were adjusted with 1 mM NaOH to pH 8.5.

We have varied the reaction parameters to control the number of oligonucleotides that is attached to each nanoparticle. This was performed as follows. First, the number of gold nanoparticles in a given dispersion of DNA‐conjugated nanoparticles was determined by atomic absorption spectroscopy (AAS), giving the gold concentration, and assuming spherical gold particles with an average diameter of 2 nm (equivalent to 250 gold atoms in each nanoparticle).^[^ ^22^ ^]^ Second, the number of attached oligonucleotides was determined by quantitative UV‐Vis spectroscopy. UV‐Vis spectra of the DNA‐functionalized gold nanoparticles showed the expected absorption bands of DNA at 260 nm and of the fluorophores Cy3 and FAM at 495 and 550 nm, respectively (Figure 2). Oligonucleotides were quantified based on the absorption of the fluorophores at 495 nm (FAM) or 550 nm (Cy3), after previously recording UV‐Vis calibration curves with the dissolved labelled oligonucleotides. The ratio of the concentrations of oligonucleotides and gold nanoparticles gave the number of oligonucleotides on each nanoparticle. Each gold nanoparticle carried about 1 to 1.5 DNA strands.

We aimed to prepare nanoparticles with only one oligonucleotide to create monovalent particles. More oligonucleotides are possible by increasing the oligonucleotide concentration during synthesis (see the experimental part). If we assume the same steric demand as with siRNA oligonucleotides, we can estimate that the maximum loading is about 6–10 oligonucleotides on each nanoparticle.^[^ ^28^ ^]^

**Figure 3:**
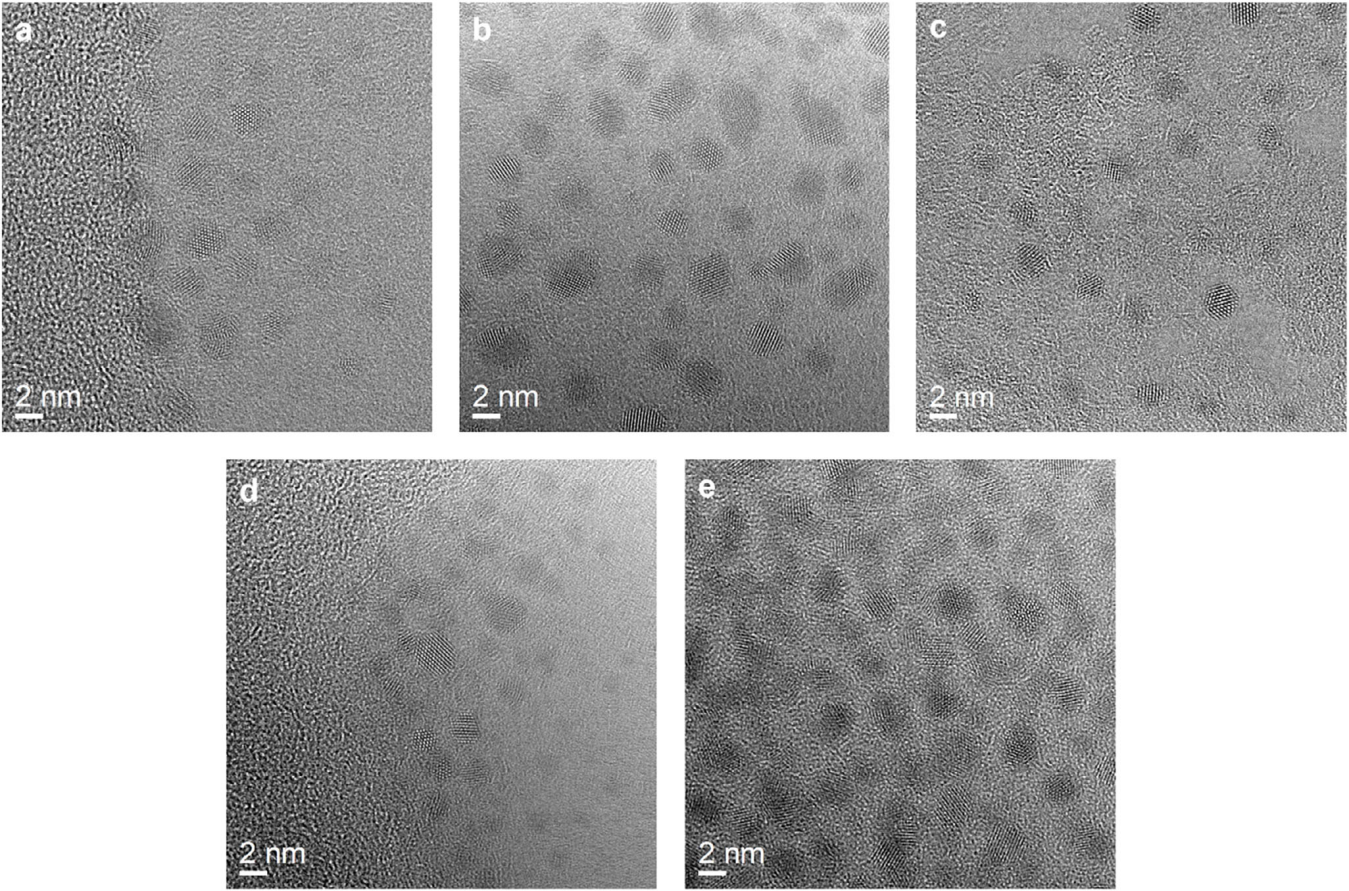
HRTEM images of the water‐dispersed DNA‐conjugated ultrasmall gold nanoparticles Au‐OligoFAM‐20 nt **a**), Au‐OligoCy3‐20 nt **b**), Au‐OligoFAM‐30 nt **c**), Au‐OligoCy3‐30 nt **d**), and Au‐OligoCy3‐30ntrev **e**).

The gold nanoparticle size before and after amine‐to‐azide conversion was determined by differential centrifugal sedimentation (DCS) and transmission electron microscopy (HRTEM). The average particle diameters by DCS were 1.5 ± 0.3 (Au‐GSH) and 1.4 ± 0.2 nm (Au‐GSH‐N_3_), respectively. By HRTEM, they were 1.3 ± 0.4 (Au‐GSH) and 1.8 ± 0.4 nm (Au‐GSH‐N_3_), respectively. These diameters are consistent with earlier results and confirm that the diameter of the metal core was not changed by the amine‐to‐azide conversion.^[^ ^22^ ^]^ DNA oligonucleotides that carried an alkyne group on one end and a fluorophore on the other end were conjugated to Au‐GSH‐N_3_ nanoparticles via copper‐catalysed azide‐alkyne cycloaddition (CuAAC; click chemistry).^[^ ^36^, ^37^ ^]^ The DNA‐conjugated nanoparticles were then studied by HRTEM and small‐angle X‐ray scattering (SAXS) to confirm that the diameter of the gold core had not changed and that no agglomeration had occurred. The fact that DCS was not able to detect the nanoparticles, i.e., that a sedimentation under these conditions was impossible, indirectly confirms the successful attachment of DNA strands. The presence of the DNA strand increases the hydrodynamic diameter and considerably lowers the sedimentation rate.^[^ ^28^ ^]^ Figure 3 shows representative HRTEM images of DNA‐conjugated gold nanoparticles.

**Figure 4:**
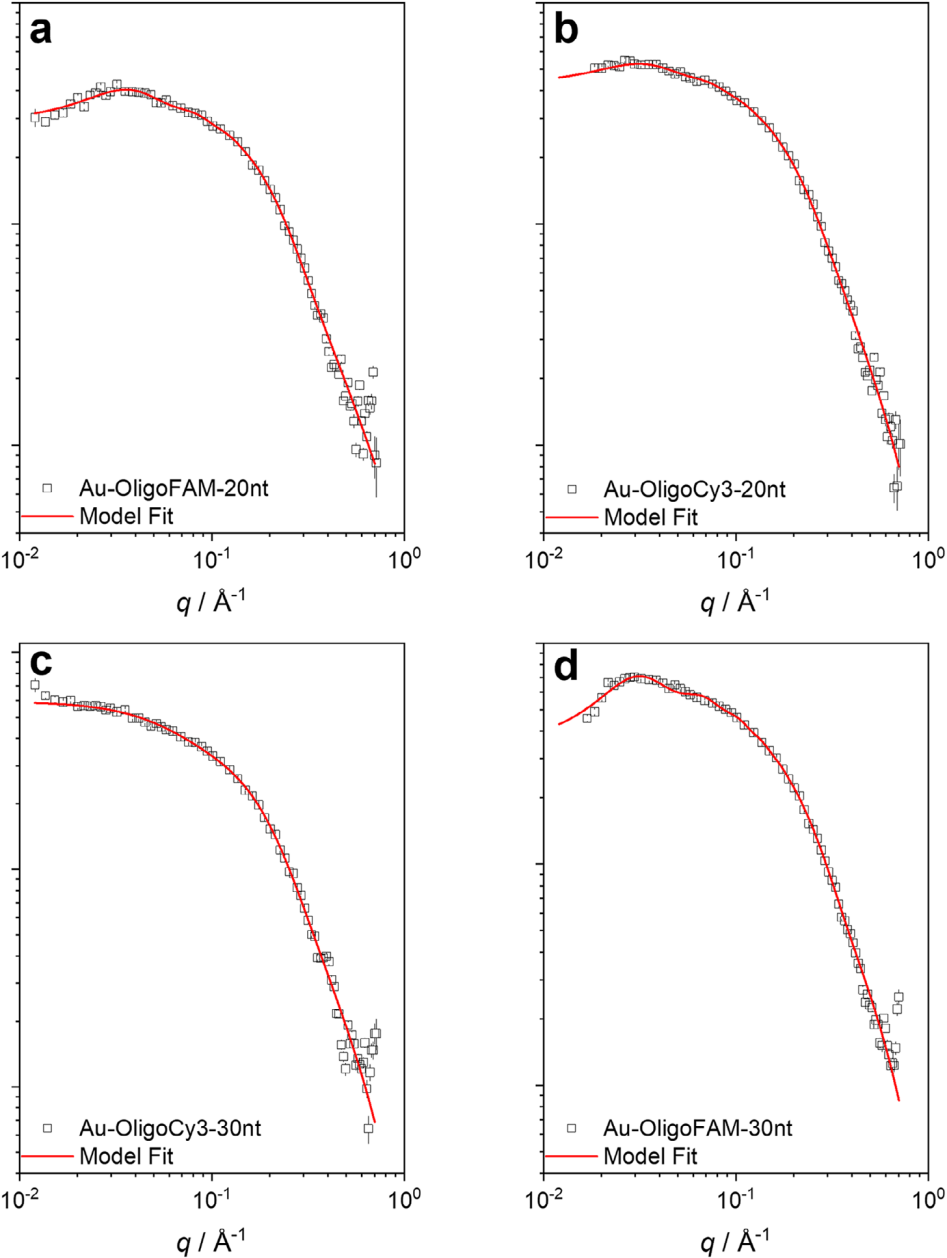
SAXS curves of the DNA‐conjugated ultrasmall gold nanoparticles Au‐OligoCy3‐20 nt **a)**, Au‐OligoFAM‐20 nt **b)**, Au‐OligoCy3‐30 nt **c)**, and Au‐OligoFAM‐30 nt **d)**. All samples were adjusted with 1 mM NaOH to pH 8.5.

Small‐angle X‐ray scattering confirmed that the particles were well dispersed in water and not agglomerated (Figure 4). The particles were modelled as hard spheres and aggregates. All particles had a diameter of ∼2 nm and a polydispersity of ∼0.2 nm. There was a tendency to form small fractions of aggregates (∼3 nm radius of gyration) and also for particle‐particle interactions, indicated by the presence of repulsive structure factor interactions. Table 2 comprises all results from SAXS.

**Table 2:** Results of small‐angle X‐ray scattering of DNA‐conjugated ultrasmall gold nanoparticles.

Sample	*d* =* 2R* / nm	*σ* / nm	*SC*_agg_	*RG*_agg_ / nm	*R*_HS_ / nm	*η*
Au‐OligoFAM‐20nt	2.2 ± 0.2	0.24 ± 0.05	0.18 ± 0.03	2.9 ± 0.6	7.3 ± 0.4	0.05 ± 0.01
Au‐OligoCy3‐20nt	2.0 ± 0.2	0.26 ± 0.05	0.19 ± 0.04	2.3 ± 0.3	8.1 ± 0.6	0.03 ± 0.01
Au‐OligoFAM‐30nt	1.2 ± 0.2	0.27 ± 0.05	0.39 ± 0.03	3.0 ± 0.3	‐	‐
Au‐OligoCy3‐30nt	2.2 ± 0.1	0.17 ± 0.06	0.37 ± 0.08	1.7 ± 0.2	9.0 ± 0.2	0.09 ± 0.01

**Figure 5:**
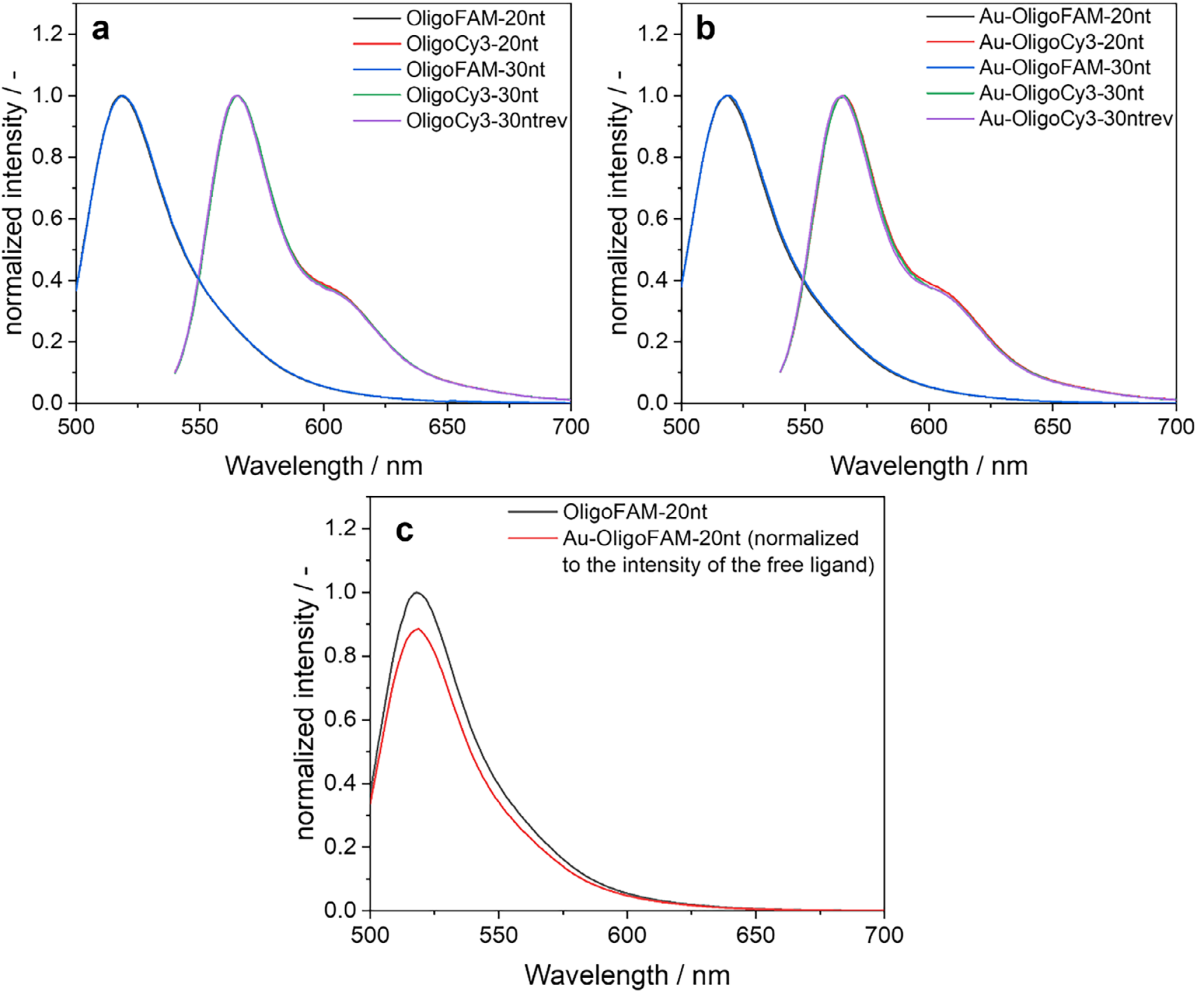
Normalized fluorescence spectra (25 °C, water) of labelled oligonucleotides before **a**) and after conjugation to gold nanoparticles **b**). The fluorescence spectra of the labelled oligonucleotides were not changed by conjugation to the nanoparticles. **c**) Comparison of the intensity of OligoFAM‐20 nt and Au‐OligoFAM‐20 nt at the same concentration of oligonucleotide showed a low degree of quenching by the vicinity of the gold core. All samples were adjusted with 1 mM NaOH to pH 8.5.

The attached dyes FAM and Cy3 showed a strong fluorescence, both in dissolved state and after conjugation to the gold nanoparticles (Figure 5). Interestingly, the degree of quenching caused by the vicinity of the metal nanoparticles was low. This can be ascribed to the ultrasmall nature of the gold nanoparticles where quenching is much less prominent than in larger plasmonic gold nanoparticles.^[^ ^20^, ^38^, ^39^, ^40^ ^]^ Furthermore, the fluorescent dye was attached to the end of the oligonucleotide that points into the dispersion, i.e., the distance between fluorophore and metal is comparatively large. The exact conformation of the DNA strands is not known, but if we assume a straight molecule, the length of a 20 nt oligonucleotide will be about 11 nm,^[^ ^41^ ^]^, and that of a 30 nt oligonucleotide about 17 nm.^[^ ^41^ ^]^

**Figure 6:**
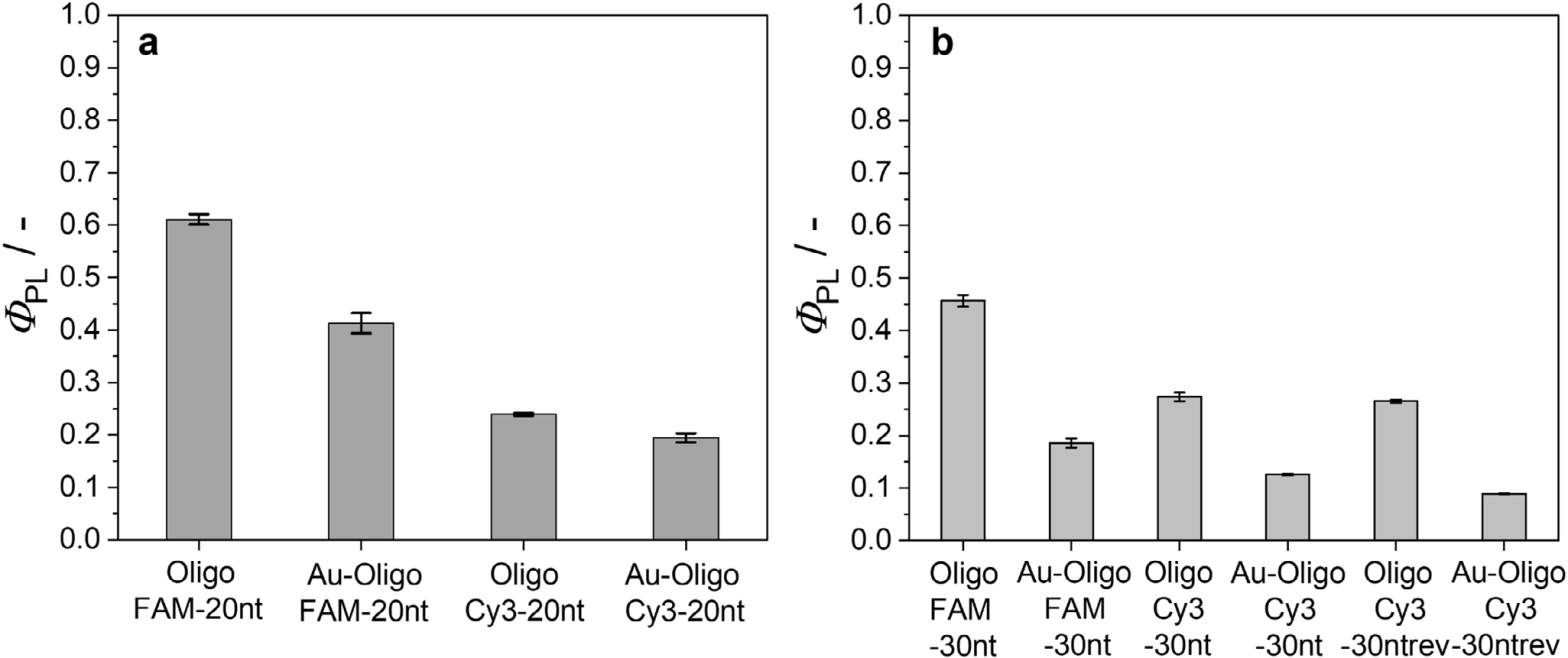
**a**) Φ_PL_ values of FAM‐ and Cy3‐labelled oligonucleotides before and after conjugation to gold nanoparticles for 20 nt **a**) and 30 nt **b**), measured at 25 °C in water. All samples were adjusted with 1 mM NaOH to pH 8.5.

The fluorescent properties can be quantitatively described by the absolute photoluminescence quantum yields *Φ*_PL_. Figure 6 shows that the presence of gold decreased *Φ*_PL_ by 20 to 50%, depending on the oligonucleotide and the fluorophore. The reported *Φ*_PL_ values for the pure dyes FAM and Cy3 are 0.93 (ref. [42]) and 0.15 (ref. [43]), respectively.

For gold nanoparticles, different quenching effects are known. In the case of larger (plasmonic) gold nanoparticles, a type of metallic surface quenching is observed, which resembles FRET but is limited to a distance of 10 nm.^[^ ^44^ ^]^ In that case, the nanoparticles act as energy acceptors, and the fluorophore transfers its excitation energy to the metal, leading to radiationless decay. Furthermore, a process denoted as Nanoparticle Surface Energy Transfer (NSET) can occur.^[^ ^45^, ^46^, ^47^ ^]^ Here, the excitation energy is directly transferred to the metal surface, where it is absorbed by surface plasmons of the nanoparticle and subsequently converted into heat. Again, the probability of fluorescence emission is significantly reduced. Unlike the FRET effect, the NSET effect can also occur over a distance of up to 30 nm. The quenching of the fluorophores on the conjugated oligonucleotides can be explained by both effects, although the effect is much smaller here than with plasmonic nanoparticles.^[^ ^48^ ^]^

**Figure 7:**
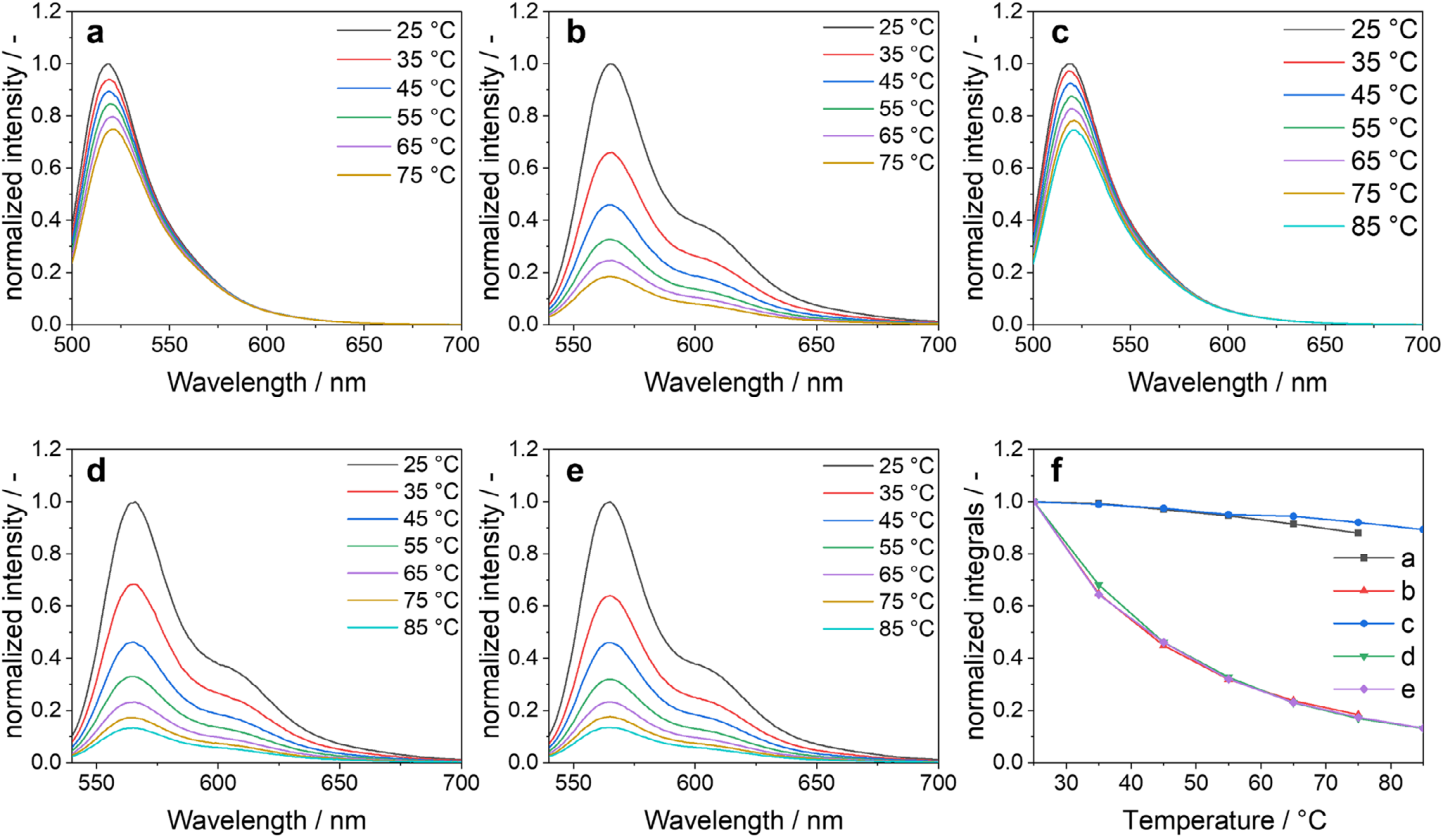
Fluorescence spectra in water at different temperature of the nanoparticles Au‐OligoFAM‐20 nt **a**), Au‐OligoCy3‐20 nt **b**), Au‐OligoFAM‐30nt **c**), Au‐OligoCy3‐30nt **d**), and Au‐OligoCy3‐30ntrev **e**). The decrease of the integrated fluorescence intensity with increasing temperature is shown **f**). All samples were adjusted with 1 mM NaOH to pH 8.5. The excitation wavelength was 490 nm for FAM and 525 nm for Cy3.

Temperature is an important parameter when hybridized oligonucleotides are studied. Dehybridization (melting) occurs at increasing temperature. We have therefore studied the effect of temperature on the fluorescence efficiency of DNA‐conjugated gold nanoparticles. In general, the fluorescence intensity decreased with increasing temperature as expected (Figure 7). A rising temperature leads to increased molecular vibrations and nonradiative relaxations, resulting in a greater conversion of energy into heat. Furthermore, the probability of collisions with solvent molecules increases.^[^ ^44^ ^]^ These collisions can cause nonradiative deactivation of the excited state (dynamic quenching).^[^ ^44^ ^]^

The significant difference in the intensity reduction can be attributed to the structural properties of the dyes. For FAM, temperature‐dependent intramolecular rotational motions induce conformational changes that facilitate nonradiative energy dissipation, thereby reducing the fluorescence.^[^ ^49^ ^]^ In contrast, the effect of temperature‐induced conformational changes is considerably more pronounced for Cy3. This fluorophore can exist in two different isomers (*cis* and *trans*), with the *trans*‐form being the fluorescent state. An increase in ambient temperature favours the conversion into the *cis*‐form, which causes a substantial decrease in fluorescence intensity.^[^ ^50^ ^]^

**Figure 8:**
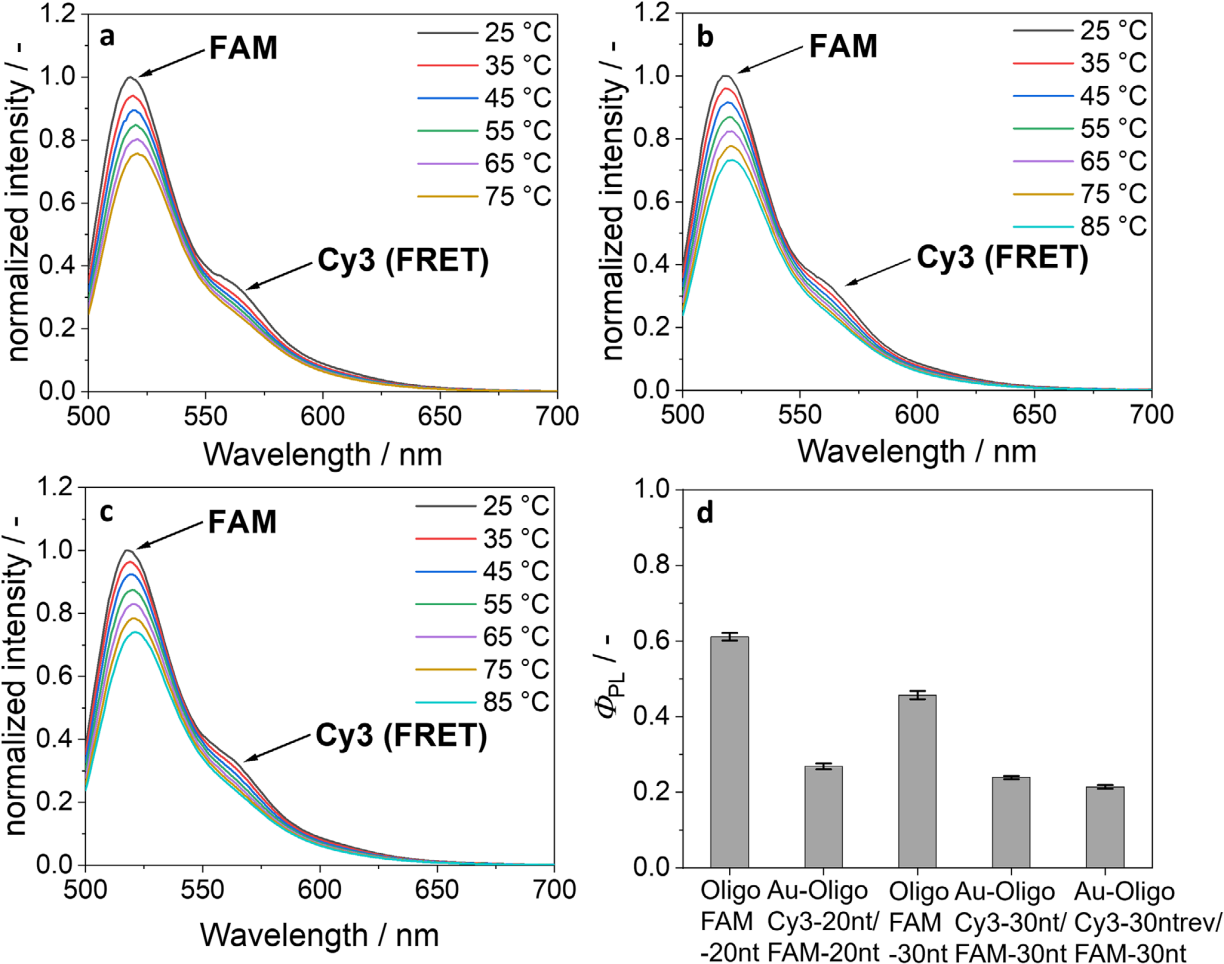
(**a‐c**) Fluorescence spectra in water at variable temperature of hybridized oligonucleotides on ultrasmall gold nanoparticles **a**) Au‐OligoCy3‐20 nt/OligoFAM‐20 nt, **b**) Au‐OligoCy3‐30 nt/OligoFAM‐30 nt, and **c**) Au‐OligoCy3‐30ntrev/OligoFAM‐30 nt. FAM was excited at 490 nm and transferred part of its excitation to Cy3 (FRET band at 565 nm) via the FRET effect. **d**) *Φ*_PL_ of hybridized oligonucleotides (water, 25 °C, excitation at 490 nm). All samples were adjusted with 1 mM NaOH to pH 8.5.

Complementary FAM‐labeled oligonucleotides were then hybridized to Au‐OligoCy3‐30 nt, Au‐OligoCy3‐20 nt, and Au‐OligoCy3‐30ntrev nanoparticles to assess the binding efficiency. The resulting double‐stranded DNA structure leads to a shortening compared to the single strand oligonucleotide. For 20 base pairs, the distance is now about 7 nm,^[^ ^51^ ^]^ and for 30 base pairs, it is about 10 nm,^[^ ^51^ ^]^ depending on the conformation. As a result, the two fluorophores come into the range for FRET of 10 nm. In our case, FRET leads to an electron transfer from a donor (FAM) to an acceptor (Cy3) because the emission spectrum of the donor overlaps with the absorption spectrum of the acceptor.^[^ ^44^ ^]^ The FRET effect was indeed found in the hybridized nanoparticles, confirming the successful conjugation. The FRET effect decreased with increasing temperature due to melting of the oligonucleotides, i.e., the separation of the two DNA strands. The presence of the nanoparticles reduced *Φ*_PL_ to approximately one half (Figure 8).

**Figure 9:**
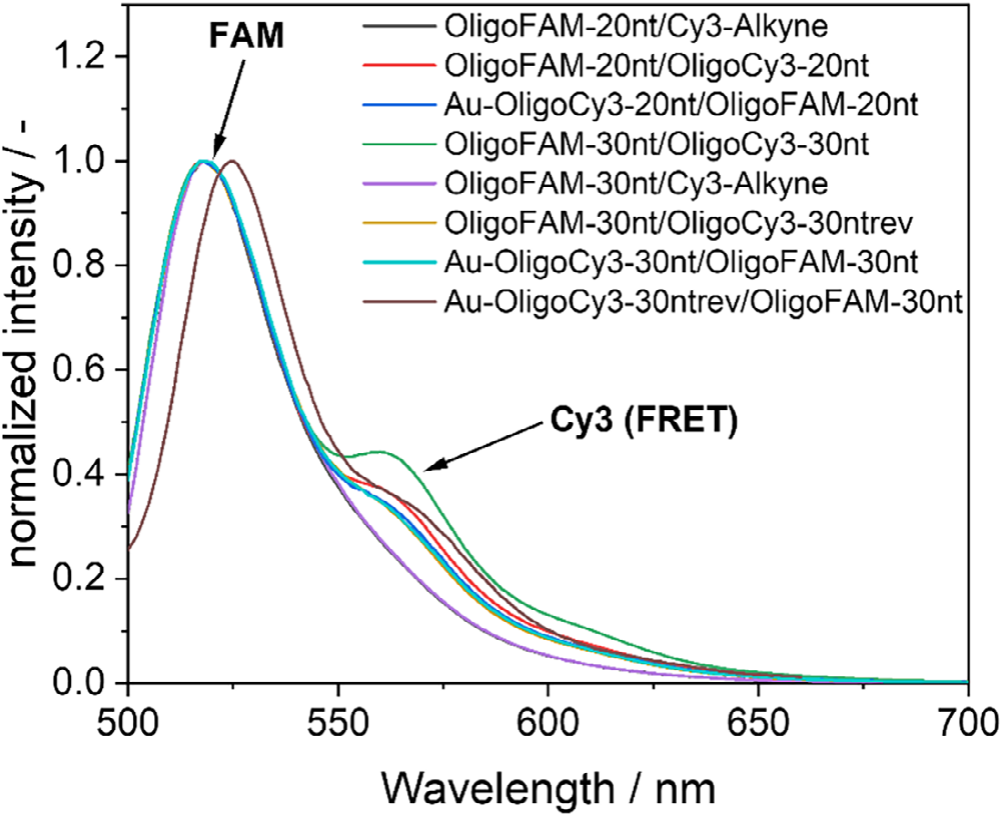
Comparison of the fluorescence intensity of hybridized DNA strands and gold‐conjugated hybridized DNA with physical mixtures of the oligonucleotides and dyes (samples OligoFAM‐20 nt/Cy3‐Alkyne and OligoFAM‐30 nt/Cy3‐Alkyne). The physical mixtures showed no FRET effect and had almost the same fluorescence curves. FAM was excited at 490 nm.

To confirm the conjugation and to exclude a purely adsorptive binding, the hybridized samples were compared with physical mixtures of the oligonucleotides and dissolved dyes. Figure 9 shows that the physical mixtures did not give a FRET effect because the fluorophores FAM and Cy3 were not in close proximity.

**Figure 10:**
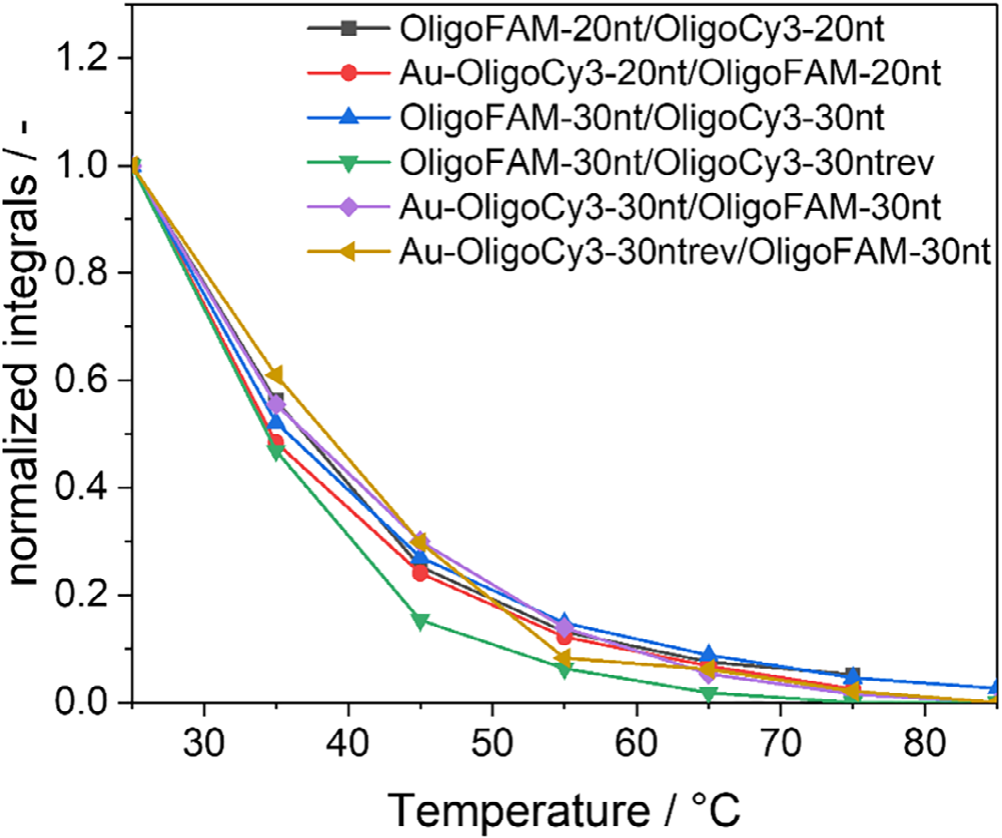
Decrease in the integrated FRET peak with increasing temperature, showing the melting of the DNA strands. All samples were adjusted with 1 mM NaOH to pH 8.5.

Figure 10 shows the decrease in the FRET effect with increasing temperature, confirming the melting and dehybridization. The monotonously decreasing FRET intensity is not directly related to the fraction of hybridized oligonucleotides because the intensity of Cy3 is itself strongly temperature‐dependent (see Figure 7). However, the curves demonstrate how the hybridization decreases with increasing temperature due to increasing thermal motion.

**Figure 11:**
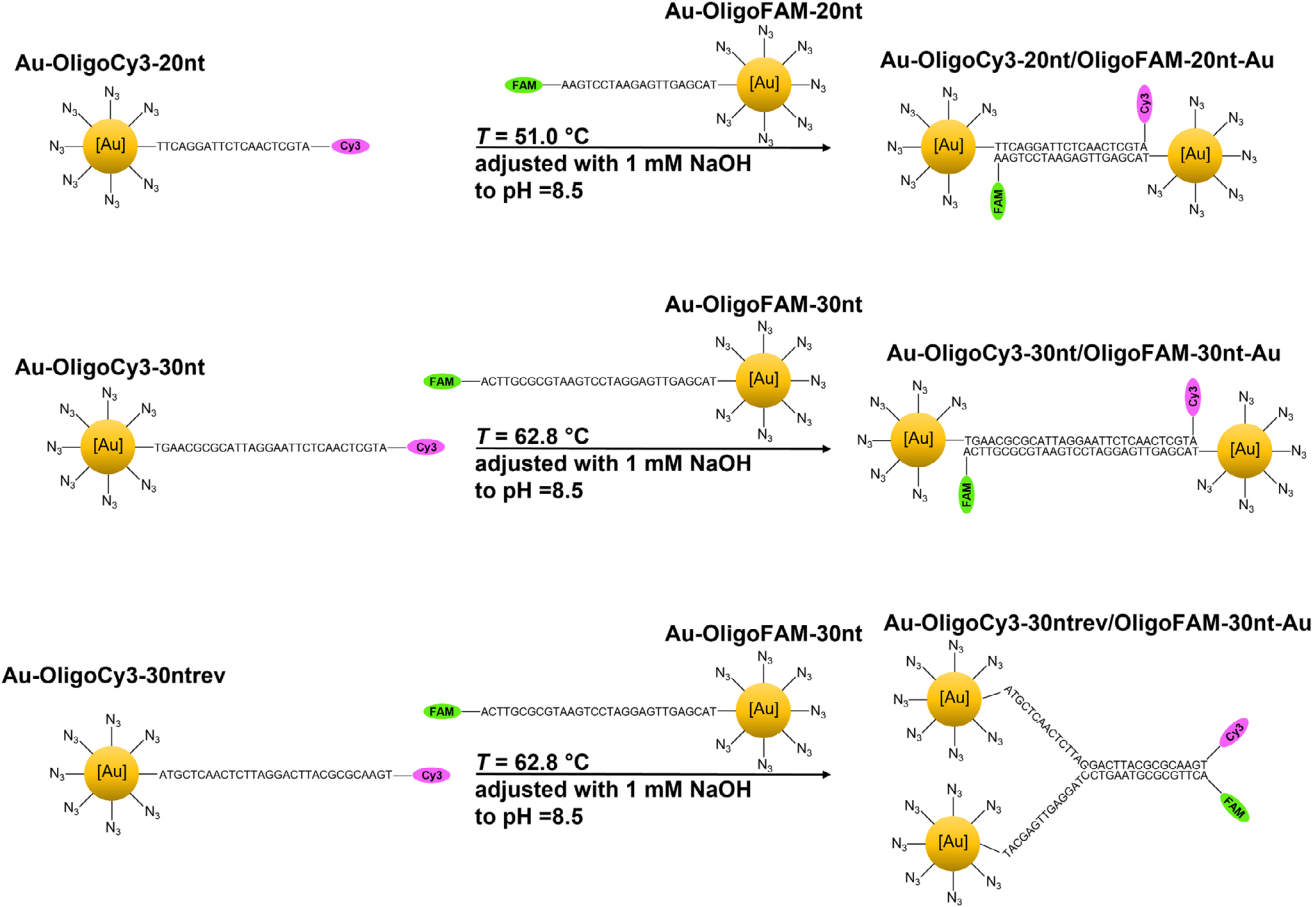
Schematic concept to prepare nanoparticle twins via DNA hybridization of gold nanoparticles that carry complementary DNA strands.

The connection of two nanoparticles by two complementary DNA strands to form nanoparticle dimers is an interesting possibility. The dimerization of plasmonic gold nanoparticles (15 nm) via click chemistry was reported earlier.^[^ ^52^, ^53^ ^]^ Figure 11 illustrates the binding concept. Of course, this is only possible if each nanoparticle carries only one DNA strand. In our case, each nanoparticle carried 1 to 1.5 DNA strands on average.

**Figure 12:**
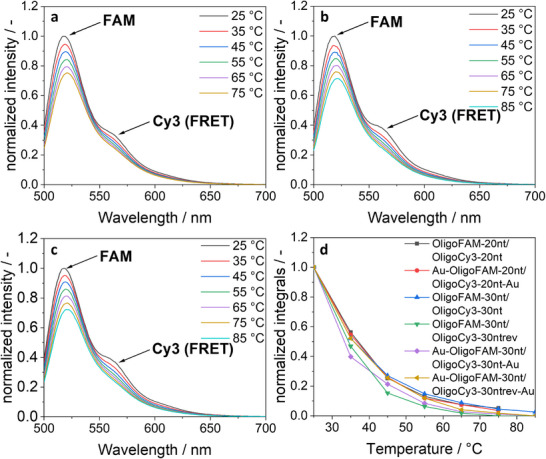
(**a‐c**) Fluorescence spectra of DNA‐connected ultrasmall gold nanoparticles in water at increasing temperature: Au‐OligoCy3‐20 nt/OligoFAM20nt‐Au **a**), Au‐OligoCy3‐30 nt/OligoFAM‐30nt‐Au **b**), and Au‐OligoCy3‐30ntrev/OligoFAM‐30nt‐Au **c**). Decrease in the FRET effect (integrated FRET band) with increasing temperature, indicating melting and dissociation of the nanoparticle pairs **d**). All samples were adjusted with 1 mM NaOH to pH 8.5.

These connected nanoparticles were also studied by temperature‐dependent fluorescence spectroscopy (Figure 12). FRET effects were again observed, indicating a successful hybridization, together with a decrease of the FRET efficiency with increasing temperature. When comparing the dissolved hybridized ligands and the nanoparticle twins, both showed a very similar behaviour in the decrease of the FRET effect with increasing temperature.

However, HRTEM and SAXS experiments indicated that the fraction of such nanoparticle dimers was actually low. Besides the expected dimers, many monomeric nanoparticles and also some aggregates of more than two nanoparticles were present. A separation of nanoparticle monomers, dimers, and multimers by gradient polyacrylamide gel electrophoresis (PAGE) was not successful, possibly due to dehybridization and nanoparticle separation inside the gel or after elution from the gel.

## 4 Conclusions

The covalent conjugation of DNA strands to ultrasmall gold nanoparticles is achievable via click chemistry. The DNA strands remain capable of hybridization with complementary sequences. Melting of the resulting DNA duplexes occurs at elevated temperature, confirming the successful prior hybridization. Although the presence of the gold core causes fluorescence quenching, the distance between the gold surface and the other end of the DNA strand is high enough to limit quenching. This also suggests that the DNA strands are not back‐folded onto the nanoparticle surface. Such DNA‐terminated ultrasmall particles can serve as effective anchoring probes for binding complementary DNA strands. In principle, it is also possible to form nanoparticle dimers by conjugating two types of nanoparticles that carry complementary DNA sequences. However, the efficiency is always small, estimated about 20–30%. The underlying reason is unclear, but it may be due to mechanical forces that tend to separate the nanoparticles, exceeding the strength of hybridization provided by a 20‐ or 30‐nucleotide DNA strand. The thermal motion of the “heavy” gold particles will increase with temperature and promote separation of the DNA strands. As a result, the hybridization is incomplete, which is underscored by the observation that a separation of monomeric, dimeric, and multimeric nanoparticles was not possible.

### 4.1 Availability of Data and Materials

All data generated or analysed during this study are included in this published article and its supplementary information files.

## Supporting Information

UV‐Vis calibration curves of oligonucleotides (Figures S1 and S2); fluorescence spectra of hybridized oligonucleotides at different temperatures (Figures S3 and S4); comparison of the fluorescence intensity of nonconjugated and gold‐conjugated oligonucleotides (Figure S5); molecular building blocks for modification of DNA according to the manufacturer (Figure S6).

## Conflict of Interest

The authors declare that they have no competing interests.

## Supporting information



Supporting Information

## Data Availability

All data generated or analysed during this study are included in this published article and its supplementary information files.
