# Supplementary material for: Hybridization on DNA‐Coated Ultrasmall Gold Nanoparticles (2 nm)
Source: Chemistry. 2025 Oct 9;31(62):e02421. doi: 10.1002/chem.202502421 (PMC12598381; doi:10.1002/chem.202502421)
Supplement: Supplementary file 1 — Supporting Information [file CHEM-31-e02421-s001.pdf]

Supporting Information

Hybridization on DNA-Coated Ultrasmall Gold Nanoparticles (2 nm)

Jonas Sager,[1] Kateryna Loza,[1] Oleg Prymak,[1] Marc Heggen,[2] Alexander Huber,[3] Jens Voskuhl,[3] Cristiano L. P. Oliveira,[4] and Matthias Epple[1,*]

[1] Inorganic Chemistry and Center for Nanointegration Duisburg-Essen (CENIDE), University of Duisburg-Essen, 45141 Essen, Germany
[2] Ernst Ruska Centre for Microscopy and Spectroscopy with Electrons, Forschungszentrum Jülich, 52428 Jülich, Germany
[3] Organic Chemistry and Center for Nanointegration Duisburg-Essen (CENIDE), University of Duisburg-Essen, 45141 Essen, Germany
[4] Institute of Physics, University of São Paulo, São Paulo 05508-090, Brazil
[*] Correspondence author: Matthias Epple, matthias.epple@uni-due.de

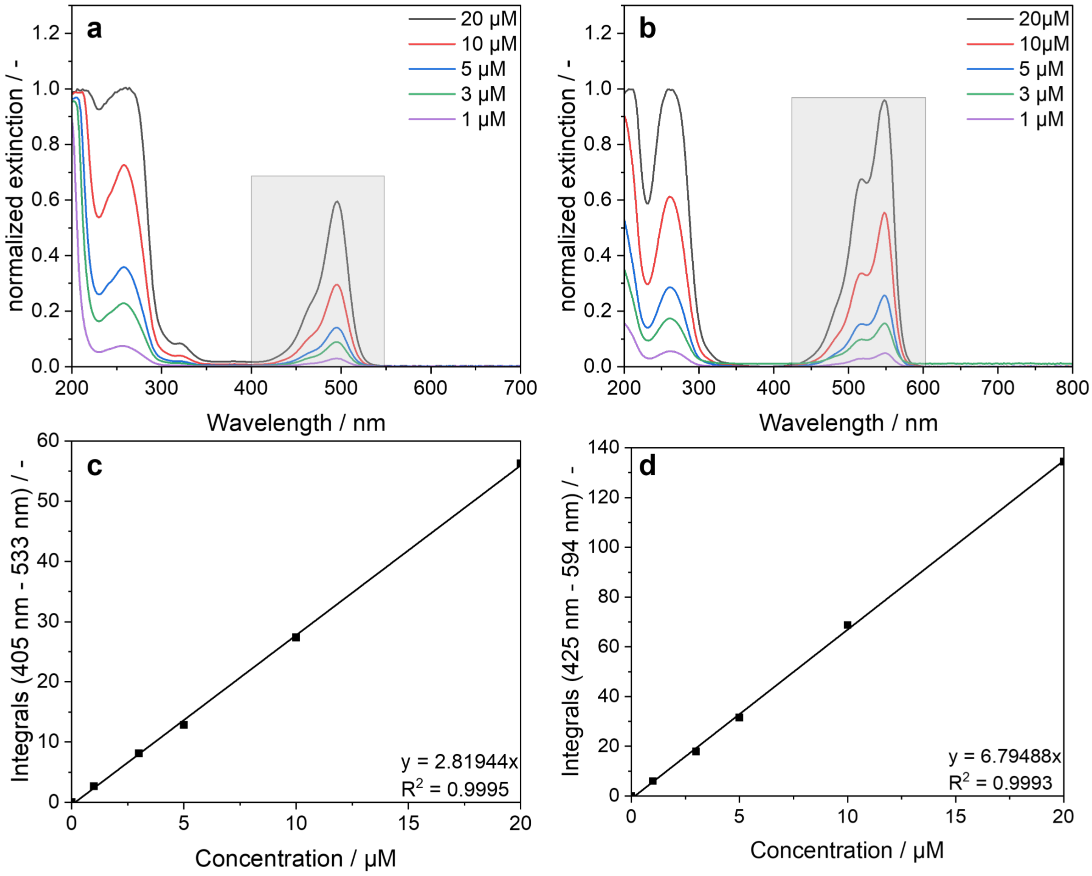

Figure S1: UV-Vis calibration curves of OligoFAM-20nt (**a**), OligoCy3-20nt (**b**), and linear regression of OligoFAM-20nt (**c**) and OligoCy3-20nt (**d**), all adjusted with 1 mM NaOH to pH 8.5.

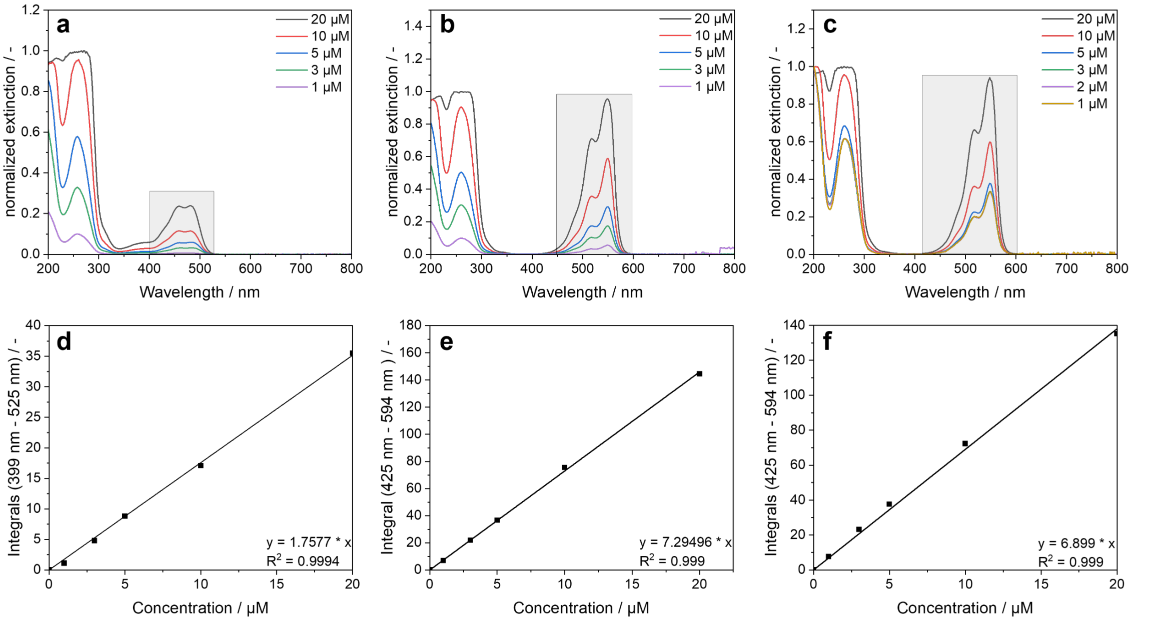

Figure S2: UV-Vis calibration curves of OligoFAM-30nt (**a**), OligoCy3-30nt (**b**), and OligoCy3-30ntrev (**c**). Linear regression of OligoFAM-30nt (**d**), OligoCy3-30nt (**e**), and OligoCy3-30ntrev (**f**), all adjusted with 1 mM NaOH to pH 8.5.

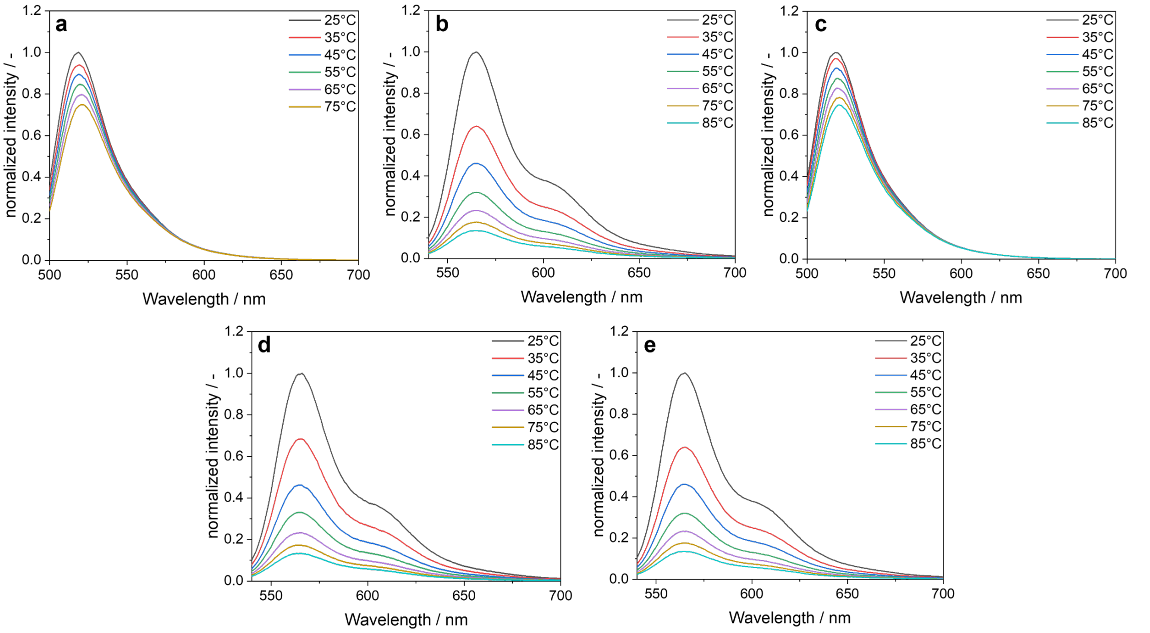

Figure S3: Fluorescence spectra at different temperatures of OligoFAM-20nt (**a**), OligoCy3-20nt (**b**) OligoFAM-30nt (**c**), OligoCy3-30nt (**d**) and OligoCy3-30ntrev (**e**). All samples were adjusted with 1 mM NaOH to pH 8.5.

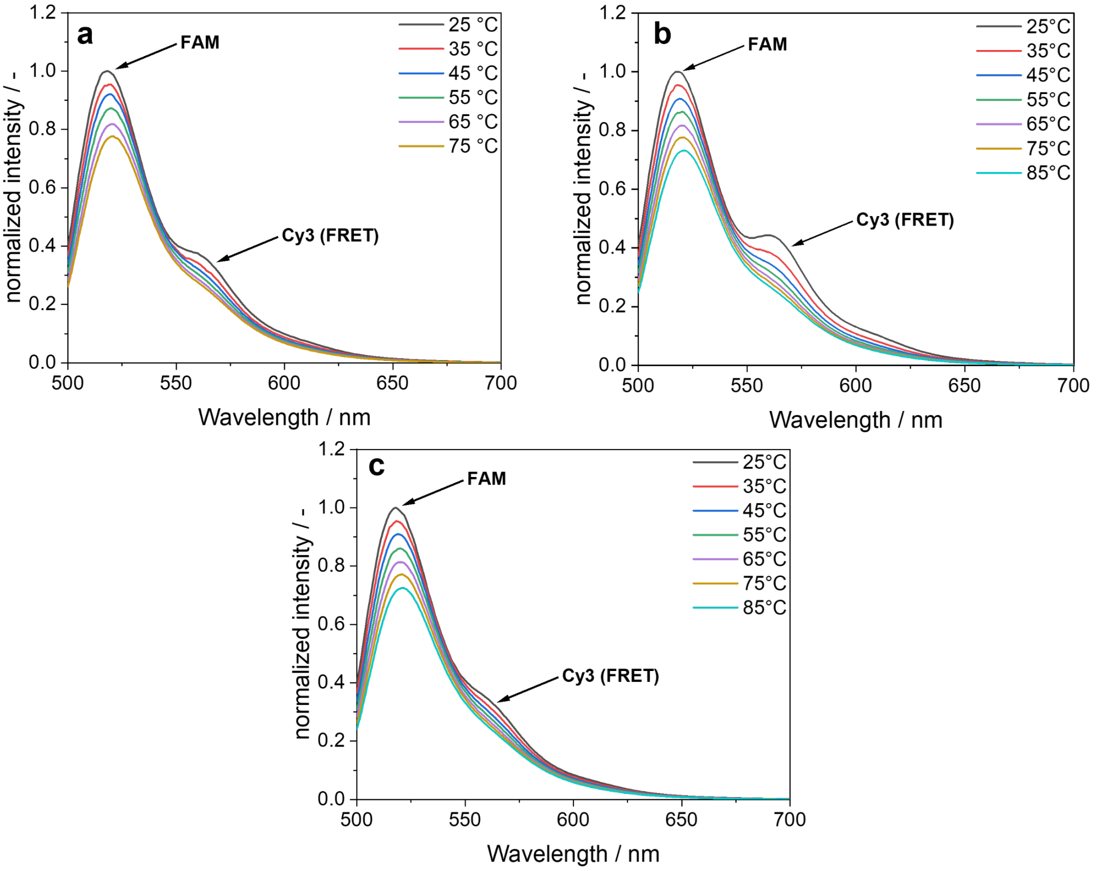

Figure S4: Fluorescence spectra at different temperatures of hybridized oligonucleotides OligoCy3-20nt/OligoFAM-20nt (**a**), OligoCy3-30nt/OligoFAM-30nt (**b**), and OligoCy3-30ntrev/OligoFAM-30nt (**c**). All samples were adjusted with 1 mM NaOH to pH 8.5.

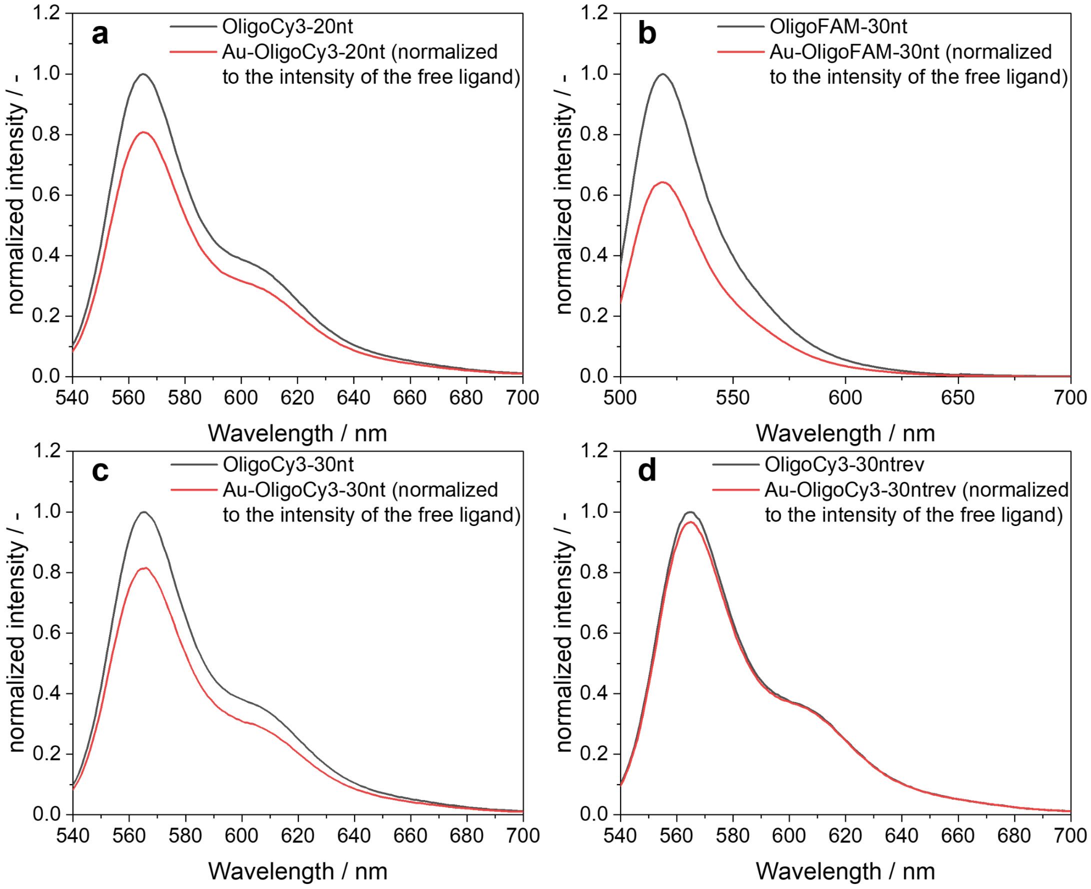

Figure S5: Comparison of the fluorescence intensity of non-conjugated and gold-conjugated oligonucleotides (**a**) OligoFAM-30nt and Au-OligoFAM-30nt, (**b**) OligoCy3-20nt and Au-OligoCy3-20nt, (**c**) OligoCy3-30nt and Au-OligoCy3-30nt and (**d**) OligoCy3-30ntrev and Au-OligoCy3-30ntrev at the same concentration of oligonucleotide. All samples were adjusted with 1 mM NaOH to pH 8.5.

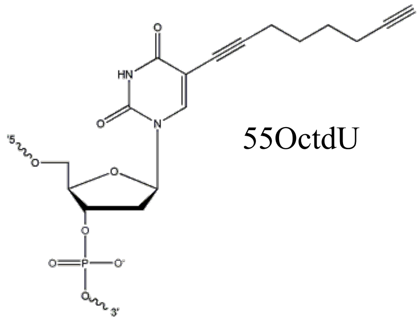

55OctdU

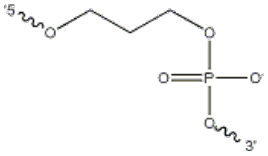

iSpC3

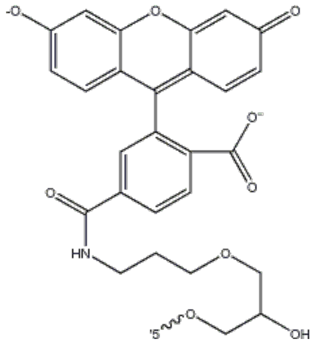

36-FAM

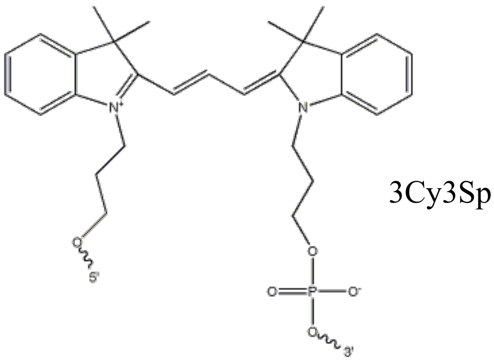

3Cy3Sp

Figure S6: Molecular building blocks for modification of DNA according to the manufacturer Integrated DNA Technologies.
